# Effects of Capacity-Related Delay to Hip Fracture Surgery on Mortality: A Matched-Cohort Study

**DOI:** 10.2106/JBJS.26.00596

**Published:** 2026-09-01

**Authors:** S.W. King, S.M. Wakefield, V.P. Giannoudis, S.D. Relton, P.V. Giannoudis, H.G. Pandit, P.L. Rodham

**Affiliations:** 1 Academic Department of Trauma and Orthopaedics, Leeds General Infirmary, Leeds, United Kingdom; 2 Leeds Institute of Rheumatic and Musculoskeletal Medicine, University of Leeds, Leeds, United Kingdom; 3 Leeds Institute for Health Sciences, University of Leeds, Leeds, United Kingdom

## Abstract

**Background:**

Timely surgery is emphasized in hip fracture care. Prior studies examining this have failed to distinguish adequately between delays for medical optimization and those due to limited operative capacity, introducing important confounders. This study aimed to determine the impact of capacity-related delays on mortality after a hip fracture in older individuals, as well as the factors moderating these effects.

**Methods:**

This retrospective cohort study involved patients treated in a U.K. teaching hospital from January 2020 to June 2024. Primary outcomes were mortality at 30 and 365 days. The exposure of interest was capacity-delayed hip fracture surgery, defined as operative anesthesia for hip fracture surgery occurring >36 hours after emergency department presentation. Patients aged ≥65 years with an operatively managed fracture of the native proximal femur were included. Patients were excluded if surgery was delayed for medical reasons. Multivariable logistic regression was used to examine mortality risk factors, and propensity-score matching was performed on the basis of covariates affecting mortality and survival analysis.

**Results:**

Of 3,094 screened patients, 2,358 were eligible. Mortality status was available for 96.9% of patients at 365 days. One thousand and eighty-seven (46%) received timely surgery while 1,271 (54%) experienced capacity-related delay. After propensity-score matching, the median age was 85 years (interquartile range, 78 to 90 years), 73% were female, and 98% were White. Capacity delay was associated with a greater hazard of 365-day mortality (hazard ratio [HR] = 1.37, 95% confidence interval [CI]: 1.15 to 1.63), and the hazard was most pronounced in the first 30 days (HR = 1.71, 95% CI: 1.11 to 2.65). Shorter-term (30-day) mortality in patients with a higher Clinical Frailty Scale score (>4) was most affected by delay (odds ratio [OR] = 1.76, 95% CI: 1.06 to 3.02), while effects on less frail patients were not significant.

**Conclusions:**

Capacity-related delays in hip fracture surgery were associated with greater mortality after accounting for patient-related risk factors. This study overcomes a key confounder by isolating capacity-related delay. The results may support prioritizing timely hip fracture surgery for more vulnerable patients.

**Level of Evidence:**

Prognostic Level III. See Instructions for Authors for a complete description of levels of evidence.

An estimated 5% to 10% of patients with a hip fracture die within 30 days of injury^1,2^, and advanced age, comorbidities, male gender, and preoperative institutional residence are mortality predictors^3^. Managing hip fractures represents a substantial health-care burden; annual institutional costs are $6 billion in the U.S.^4^. Early surgical management is a central principle, and appears associated with lower morbidity and mortality^5^. However, the definition of “early surgery” remains controversial. Most national guidelines recommend surgery within 24 to 48 hours of hospital admission^6-9^. Achievement of timely surgery varies markedly among nations and institutions^2,10^.

Medical causes of delays in surgical management typically involve comorbidities requiring optimization, while capacity-related causes reflect inadequate operating theater or surgeon capacity^11,12^. Prior literature reporting the impacts of delays on mortality has rarely separated patients delayed for medical reasons from those delayed by capacity^13^.

Services must manage base-level and surge capacity. When this fails, prioritization may be necessary. Tools such as the Nottingham Hip Fracture Score (NHFS) and Clinical Frailty Scale (CFS) may highlight patients who stand to gain the most benefit from early surgery^14-17^.

The primary aim of this study was to assess the effect of capacity-related delays to hip fracture surgery on 30- and 365-day mortality. Secondary aims were to assess the effects of capacity-related operative delay on institutional resource utilization and to identify patients disproportionately affected by operative delay, by performing an exploratory analysis using validated clinical risk tools to investigate the factors moderating these effects.

## Materials and Methods

This retrospective cohort study analyzed all patients with a fracture of the native proximal femur presenting to a U.K. teaching hospital and operatively managed between January 1, 2020, and June 30, 2024, with final follow-up occurring by October 1, 2025. Local institutional approval was obtained. Patients who were <65 years old, underwent nonoperative management, were already an inpatient at the time of fracture, or presented with a pathological fracture were excluded, consistent with previous studies^18,19^.

Data were collected for patient- and treatment-related factors by researchers blinded to mortality and other outcomes. Patient variables included the components of the NHFS^17^, American Society of Anesthesiologists (ASA) grade, CFS^16^, Index of Deprivation, fracture type, and pre-fracture mobility. The CFS was assessed by a geriatric physician on initial review. Treatment factors included the calendar year and weekday of presentation, time to geriatrician review, time in the emergency department (ED), time to the operating theater, operation performed, and anesthesia type. Ethnicity data were patient-defined, adhering to U.K. Office for National Statistics classification standards.

The reason for delay beyond 36 hours was recorded contemporaneously as part of standard care. Medical reasons included awaiting medical review, stabilization, or investigation; awaiting diagnostics; or reversal of anticoagulation therapy. Capacity reasons related to lack of operating theater capacity. To control for case complexity, patients delayed because of specialist surgical personnel or equipment requirements were excluded. The exposure of interest was the time from ED presentation to surgery. “Timely” surgery was defined as operative anesthesia commencement within 36 hours of presentation, and surgery was defined as “capacity-delayed” when anesthesia commenced after this.

### Statistical Analysis

Analysis was performed in the RStudio statistical environment (version 2025.05.1+513; Posit) utilizing packages “logistf,” “MatchIt,” “survminer,” and “cobalt.” Complete-case analysis was performed. Significance was set at p < 0.05. Unadjusted outcomes for delayed patients were compared between those delayed for medical and capacity reasons; patients delayed for medical reasons were then excluded.

Multivariable analysis to identify factors affecting mortality was performed by fitting Firth-penalized logistic regression models to estimate adjusted odds ratios (aORs) for 30- and 365-day all-cause mortality. Factors were selected on the basis of their reported association with mortality in the literature^14,20-22^. As a sensitivity analysis, bootstrap confidence intervals (CIs) were estimated for aORs by resampling the complete-case analytic cohort with replacement (1,000 replicates) and refitting the same model in each.

Propensity scores were estimated with logistic regression, with timely surgery within 36 hours as the exposure. Other predefined covariates included age, sex, preoperative hemoglobin, confusion on presentation, residence before admission, presence of ≥2 comorbidities, and active malignancy within the previous 20 years; these match the variables defined in the NHFS, a scoring system that has been shown to predict mortality^17^. Covariates to control for variations due to resource availability and implant choice were the year of presentation, weekday versus weekend presentation, fracture type, and pre-fracture mobility^8^. Continuous covariates were entered as linear terms. Missing continuous covariates were imputed as the median, and missing categorical covariates were handled using an explicit “missing” category.

Patients were matched 1:1 using nearest-neighbor matching (caliper, 0.1 times the standard deviation [SD]). The Mahalanobis distance was utilized for age, hemoglobin, and comorbidities during the matching process to optimize balance on key prognostic factors. Balance was subsequently confirmed using standardized mean differences (SMDs). Kaplan Meier 365-day survival analysis was performed. Cox proportional-hazards modeling with robust standard errors clustered by matched pairs was used to estimate the hazard ratio (HR) of 1-year mortality for capacity-related delay beyond 36 hours versus timely surgery. Post hoc piecewise Cox models over 0 to 30 days and 31 to 365 days were also fitted separately, in keeping with periods commonly used in the hip fracture literature. Two sensitivity analyses were performed. First, a 48-hour cutoff was examined, given the international variations in definitions of timely surgery. Second, the potential influences of outliers operated on very early (<12 hours) or late (>60 hours) were eliminated by comparing 24-hour windows on either side of the 36-hour cutoff (12 to 36 hours and >36 to 60 hours), thus simulating the effects of waiting an additional day preoperatively.

Finally, an exploratory analysis of low- versus high-risk patients was undertaken. Previous authors have described an NHFS of ≥6 as “high risk”; therefore, patients were divided into 2 groups (NHFS of 0 to 5 and 6 to 10) on the basis of this threshold^23^. For each group, 30- and 365-day mortality rates were calculated according to whether or not surgery occurred within 36 hours. Differences between the timely and delayed groups were assessed using logistic regression models to estimate ORs with 95% CIs. The analysis was repeated using the CFS, for which a score of ≥5 is the generally accepted definition of frailty^24^.

## Results

### Patient Selection

Of the 3,397 patients who presented with a femoral fracture during the study period, 1,039 did not meet the inclusion criteria and were excluded; 392 of these had been delayed for medical reasons and 6 had been delayed until a specialist surgeon or required equipment became available (Fig. 1). Medically-delayed patients had worse risk profiles and greater postoperative mortality than those delayed for capacity reasons (30-day: 13% versus 4.5%, p < 0.001) (see Appendix Tables 1 and 2). The remaining 2,358 patients with a hip fracture formed the study cohort; 1,087 (46%) had timely surgery and 1,271 (54%) experienced a capacity-related delay. Causes of capacity-related delays are quantified in Appendix Table 3. Baseline characteristics of the unmatched timely and capacity-delayed cohorts are summarized in Appendix Table 4, and perioperative care is described in Appendix Table 5. Before adjustment, mortality was greater in the capacity-delayed group at both 30 days (4.5% versus 2.9%) and 365 days (28% versus 23%) (see Appendix Table 6). Loss to follow-up was 0.9% at 30 days and 3.1% at 365 days.

**Fig. 1:**
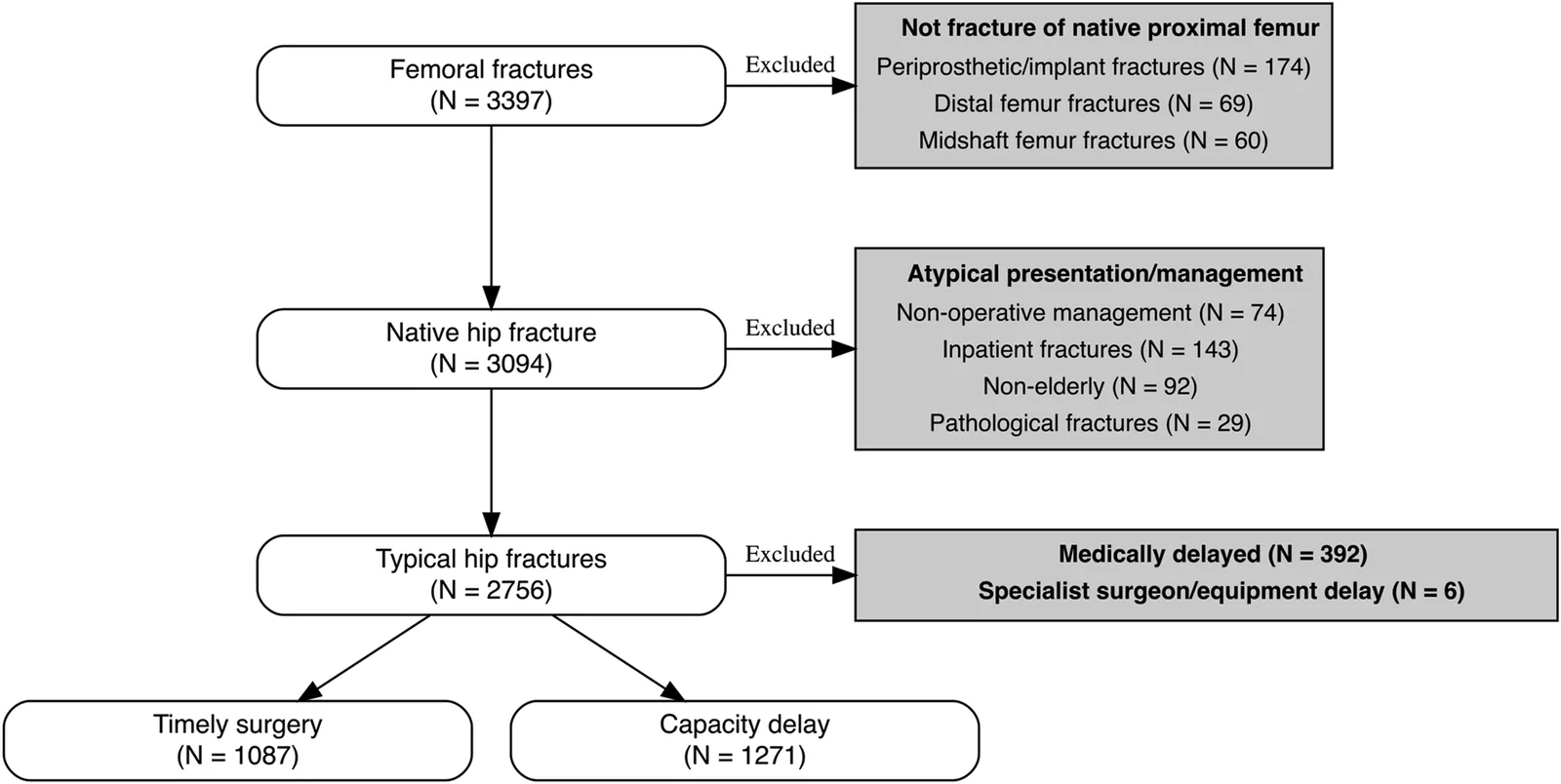
Flowchart for inclusion of patients with a hip fracture.

### Adjusted Mortality Analysis

Results are displayed in Appendix Tables 7 and 8. After adjusting for confounders, capacity-related delay beyond 36 hours was associated with greater 30-day mortality (aOR = 1.76, 95% CI: 1.07 to 2.95; p = 0.025) and 365-day mortality (aOR = 1.28, 95% CI: 1.02 to 1.62; p = 0.037). Age of ≥86 years, male sex, higher ASA class, higher CFS, hemoglobin of <100 g/L, residence in institutional care, and confusion on presentation were also associated with greater adjusted odds of mortality. Sensitivity analyses demonstrated consistency with the primary findings.

### Propensity Score-Matched Mortality Analysis

Propensity-score matching yielded 955 matched pairs. Covariate balance was improved after matching, with all SMDs for the prespecified covariates being less than the a priori threshold of 0.1 (see Appendix Fig. 1).

Baseline demographics for the propensity score-matched cohort are shown in Table 1, and clinical outcomes are detailed in Table 2. Capacity-delayed surgery was associated with greater 30-day mortality compared with timely surgery (5.2% versus 3.1%, p = 0.026) and greater 365-day mortality (27% versus 21%, p = 0.0013). Kaplan-Meier survival curves are shown in Figure 2. In the matched analysis, survival differed by operative timing (log-rank p = 0.00076). Patients undergoing capacity-delayed surgery experienced a longer hospital stay (17 versus 15 days, p < 0.001) and incurred greater median acute inpatient costs (£17,931 versus £16,716, p < 0.001).

**Table 1:** Baseline Characteristics of Matched Patients Undergoing Timely Versus Capacity-Delayed (>36 Hours) Surgery After Hip Fracture*

Characteristic	Overall (N = 1,910)†	Timing of Surgery		P Value
		Timely (N = 955)†	Capacity-Delayed (N = 955)†	
Age *(yr)*	85 [78, 90]	85 [78, 90]	85 [78, 90]	0.469‡
Sex				0.959§
Male	521 (27%)	261 (27%)	260 (27%)	
Female	1,389 (73%)	694 (73%)	695 (73%)	
Ethnicity				0.888#
Asian or Asian British	29 (1.6%)	17 (1.8%)	12 (1.3%)	
Black or Black British	4 (0.2%)	2 (0.2%)	2 (0.2%)	
Mixed or other ethnicity	7 (0.4%)	3 (0.3%)	4 (0.4%)	
White	1,801 (98%)	901 (98%)	900 (98%)	
Missing	69	32	37	
Fracture type				0.856§
Intracapsular	1,090 (57%)	539 (56%)	551 (58%)	
Per- or intertrochanteric	724 (38%)	367 (38%)	357 (37%)	
Subtrochanteric	96 (5.0%)	49 (5.1%)	47 (4.9%)	
ASA grade				0.086§
1-2	304 (17%)	165 (18%)	139 (15%)	
3-5	1,526 (83%)	746 (82%)	780 (85%)	
Missing	80	44	36	
Preoperative hemoglobin *(g/L)*	125 [113, 136]	125 [114, 136]	124 [113, 136]	0.340‡
Missing	3	1	2	
Nottingham Hip Fracture Score				0.167§
0-5	1,015 (54%)	523 (55%)	492 (52%)	
6-10	873 (46%)	422 (45%)	451 (48%)	
Missing	22	10	12	
Clinical Frailty Scale score				0.358§
1-4	787 (41%)	404 (42%)	383 (40%)	
5-9	1,118 (59%)	550 (58%)	568 (60%)	
Missing	5	1	4	
Confused on presentation	662 (35%)	328 (35%)	334 (35%)	0.759§
Missing	19	9	10	
Pre-fracture mobility				0.972#
Freely mobile without aids	690 (37%)	348 (37%)	342 (36%)	
Mobile outdoors with 1 aid	399 (21%)	201 (21%)	198 (21%)	
Mobile outdoors with 2 aids or frame	385 (20%)	186 (20%)	199 (21%)	
Some indoor mobility	407 (22%)	205 (22%)	202 (21%)	
No functional mobility	6 (0.3%)	3 (0.3%)	3 (0.3%)	
Missing	23	12	11	
Residence before admission				0.707§
Own home	1,526 (80%)	760 (80%)	766 (80%)	
Residential care	111 (5.8%)	53 (5.5%)	58 (6.1%)	
Nursing care	273 (14%)	142 (15%)	131 (14%)	
Index of Deprivation quintile				0.946§
Q1 (most deprived)	594 (31%)	294 (31%)	300 (32%)	
Q2	224 (12%)	117 (12%)	107 (11%)	
Q3	334 (18%)	167 (18%)	167 (18%)	
Q4	438 (23%)	222 (23%)	216 (23%)	
Q5 (least deprived)	309 (16%)	151 (16%)	158 (17%)	
Missing	11	4	7	

* ASA = American Society of Anesthesiologists.

† Values are given as the median [interquartile range], number (%), or number.

‡ Wilcoxon rank-sum test.

§ Chi-square test.

# Fisher exact test.

**Table 2:** Outcomes of Matched Patients Undergoing Timely Versus Capacity-Delayed (>36 Hours) Surgery After Hip Fracture

Outcome	Timing of Surgery		P Value
	Timely (N = 955)*	Capacity-Delayed (N = 955)*	
Mortality by 30 days	29 (3.1%)	49 (5.2%)	0.026†
Missing	5	11	
Mortality by 1 year	204 (21%)	262 (27%)	0.0013†
Missing	21	33	
Acute inpatient cost *(£)*	16,716 [12,355, 24,482]	17,931 [13,630, 24,771]	<0.001‡
Missing	1	2	
Length of stay *(days)*	15 [11, 22]	17 [13, 25]	<0.001‡
Missing	8	17	
Acute discharge destination			0.059†
Own home or sheltered housing	276 (29%)	268 (29%)	
Institutional care	135 (14%)	123 (13%)	
Rehabilitation unit	409 (43%)	383 (41%)	
Other acute hospital	12 (1.3%)	10 (1.1%)	
Died before acute discharge	29 (3.1%)	55 (5.9%)	
Other	86 (9.1%)	99 (11%)	
Missing	8	17	

* Values are given as the median [interquartile range], number (%), or number.

† Chi-square test.

‡ Wilcoxon rank-sum test.

**Fig. 2:**
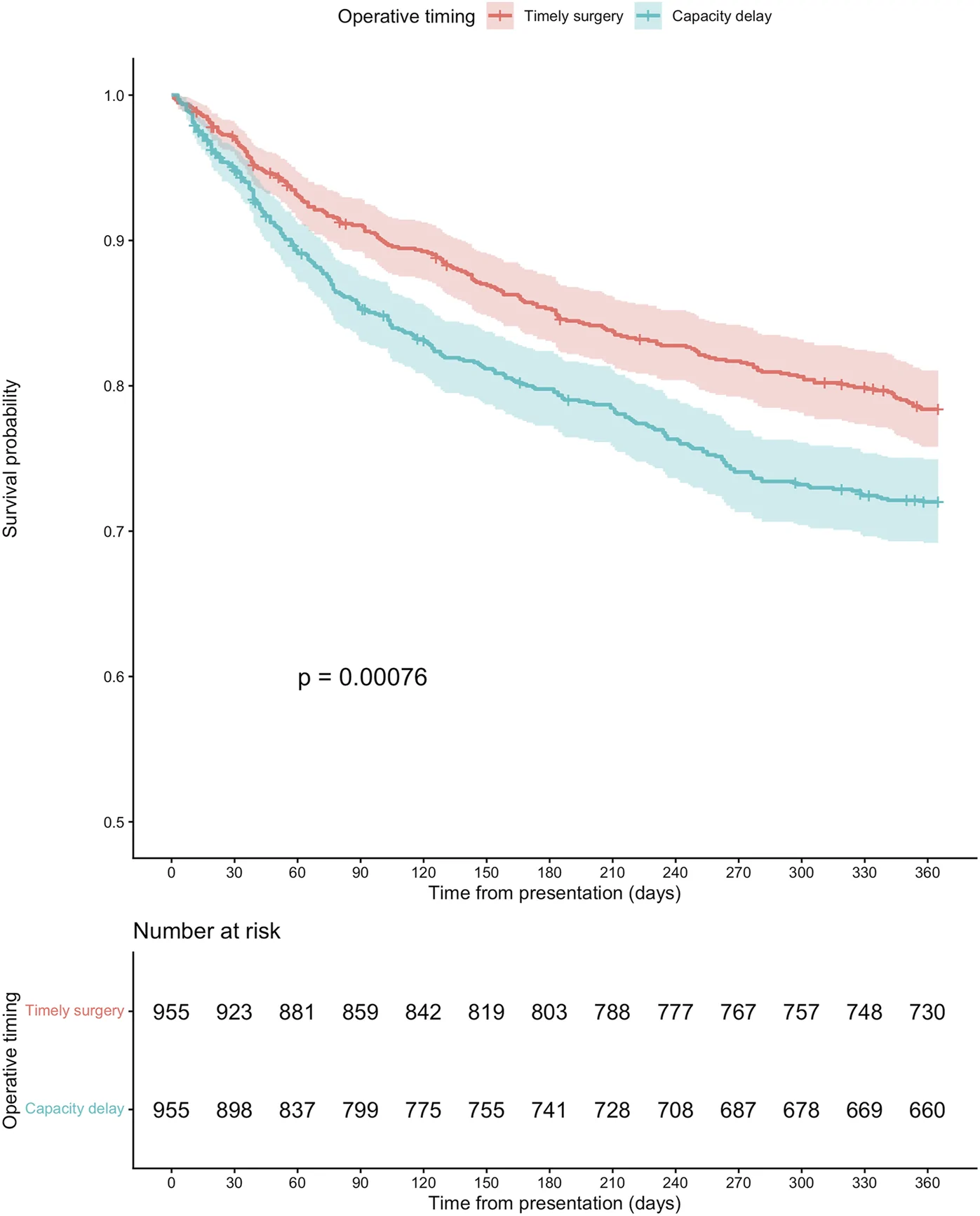
Kaplan-Meier curves for survival over the first year after a hip fracture by operative timing status in the matched cohorts. Shading indicates the 95% confidence interval.

In Cox proportional-hazards modeling, capacity-related delay beyond 36 hours was associated with greater all-cause 365-day mortality compared with timely surgery (HR = 1.37, 95% CI: 1.15 to 1.63; p < 0.001). Piecewise Cox models demonstrated that the excess hazard was greater within the first 30 days (HR = 1.71, 95% CI: 1.11 to 2.65; p = 0.016) but also persisted during days 31 to 365 (HR = 1.31, 95% CI: 1.07 to 1.60; p = 0.0076).

A 48-hour cutoff also demonstrated greater 365-day mortality in patients with capacity delay (HR = 1.47, 95% CI: 1.19 to 1.81; p < 0.001). A piecewise Cox model did not reach significance for the first 30 days (HR = 1.63, 95% CI: 0.96 to 2.78; p = 0.073). Restricting the cohort to those with times within a 24-hour window on either side of the 36-hour cutoff again demonstrated greater 365-day mortality (HR = 1.26, 95% CI: 1.04 to 1.53; p = 0.017). A piecewise Cox model did not reach significance for the first 30 days (HR = 1.40, 95% CI: 0.88 to 2.25; p = 0.16).

### Patient Risk Stratification

#### Nottingham Hip Fracture Score

The median NHFS was 5; 1,254 patients had an NHFS of 0 to 5 and 1,072 had an NHFS of 6 to 10, with the remaining 32 patients having a missing NHFS. Delay was associated with greater mortality in patients with a higher NHFS than in those with a lower NHFS (Figs. 3-A and 3-B). At 30 days, mortality in patients with an NHFS of 6 to 10 was 4.9% (95% CI: 3.3% to 7.3%) after timely surgery versus 7.8% (95% CI: 5.8% to 10.4%) after delayed surgery (OR = 1.63, 95% CI: 0.99 to 2.74; p = 0.0612). At 365-day follow-up in the same group, mortality was 38.4% (95% CI: 34.1% to 43.0%) when surgery was timely versus 46.1% (95% CI: 42.0% to 50.3%) when it was delayed (OR = 1.37, 95% CI: 1.07 to 1.76; p = 0.0122). Among patients with an NHFS of 0 to 5, no significant mortality difference was observed between timely and delayed surgery at either interval.

**Fig. 3:**
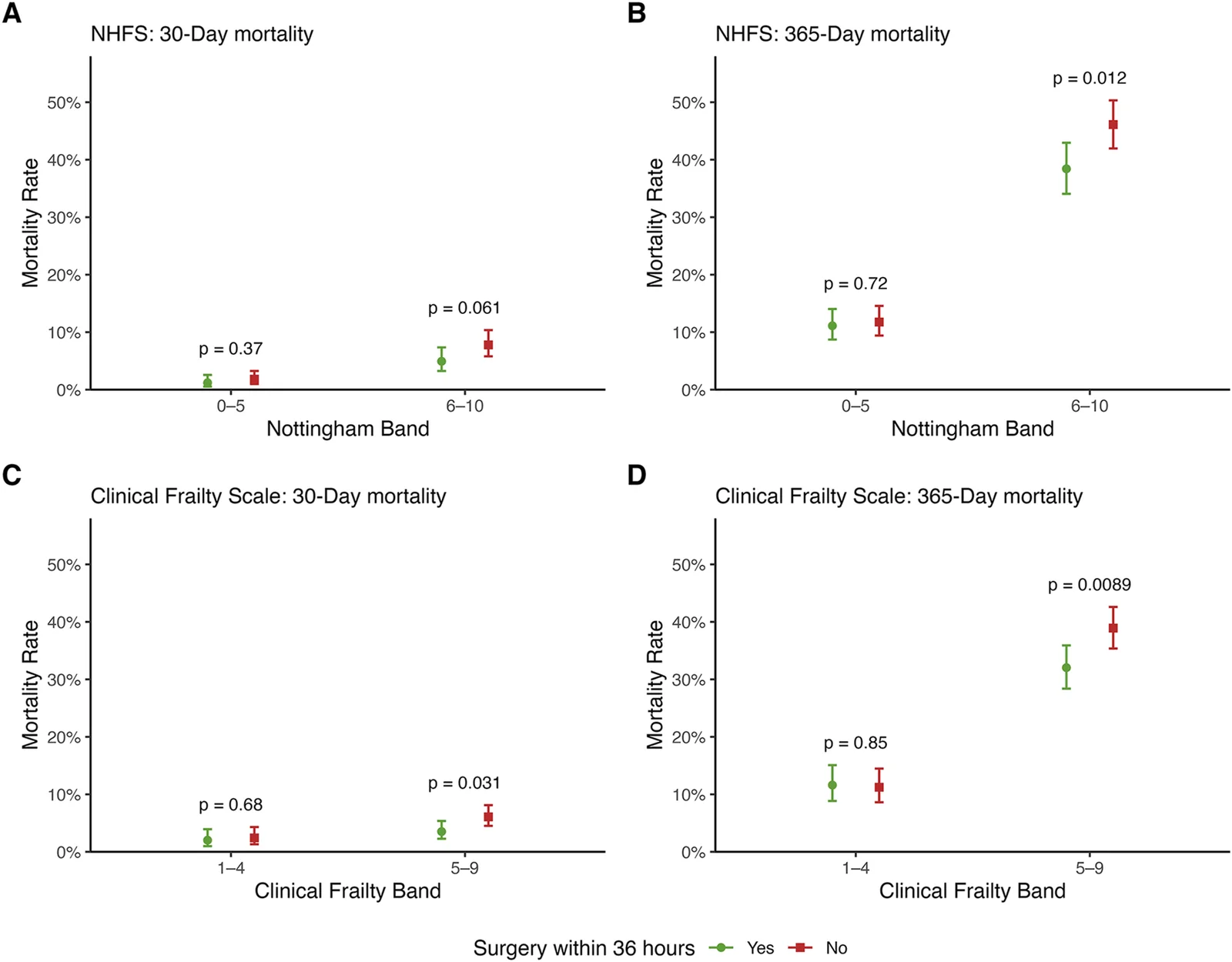
Comparison of mortality rates for timely versus delayed surgery, stratified by risk score. The error bars indicate the 95% confidence interval. **Fig. 3-A:** Comparisons of 30-day mortality stratified by Nottingham Hip Fracture Score (NHFS) group. **Fig. 3-B:** Comparisons of 365-day mortality stratified by Nottingham Hip Fracture Score group. **Fig. 3-C:** Comparisons of 30-day mortality stratified by Clinical Frailty Scale group. **Fig. 3-D:** Comparisons of 365-day mortality stratified by Clinical Frailty Scale group.

#### Clinical Frailty Scale

The median CFS was 5. There were 965 patients with a CFS of 1 to 4 and 1,388 with a CFS of 5 to 9; the CFS was missing for the remaining 5 patients. At 30 days, mortality among the frailer patients was 3.5% (95% CI: 2.3% to 5.3%) after timely surgery versus 6.0% (95% CI: 4.5% to 8.0%) after delayed surgery (OR = 1.76, 95% CI: 1.06 to 3.02; p = 0.031) (Figs. 3-C and 3-D). At 365 days, mortality in the frailer group was 31.9% (95% CI: 28.3% to 35.7%) after timely surgery versus 38.4% (95% CI: 34.9% to 42.0%) after delayed surgery (OR = 1.33, 95% CI: 1.06 to 1.67; p = 0.0089). In the less frail group, no significant mortality difference was observed between timely and delayed surgery at either interval.

## Discussion

This study demonstrated that capacity delays were independently associated with greater mortality risk, inpatient stay, and institutional costs, identifying them as a primary target for service optimization. Although medical delays are often patient-inherent, capacity delays are potentially modifiable system factors.

Most prior literature is methodologically limited. Numerous authors have demonstrated greater mortality with operative delay but failed to account for the causes of the delay, limiting causal inference^18,25-28^. The current study demonstrated that patients delayed for medical reasons had higher ASA grades and NHFS, and were almost threefold more likely to die in the first 30 days after presentation compared with capacity-delayed patients, highlighting the importance of such differentiation between delay causes^13^. When authors fail to account for the delay reason, it is not possible to conclude whether the inferior outcome in the delayed group was caused by surgical timing or a poorer medical condition that predisposes to greater all-cause mortality.

A recent study that did adjust for the delay reason found no difference in mortality between timely and delayed surgery, but it had only 375 patients in the delayed group and so was likely underpowered^19^. A U.K. study reported greater mortality in patients delayed >12 hours for nonmedical reasons, but that study period spanned 30 years^29^. The current study excluded patients delayed for medical optimization, overcoming a common confounder limiting previous studies. Its propensity-score matching accounted for both risk factors for mortality and disparities in health-care capacity, further increasing the strength of our study’s conclusions.

Factors affecting the delivery of hip fracture care can be complex^30^. Evidence-based recommendations for improving such care include dedicated 7-day hip fracture trauma rooms, senior-led surgery and anesthesia, daily multidisciplinary trauma meetings, fast-track pathways, and formal escalation plans to generate surge capacity^31,32^. The greater costs incurred by delays provide financial justification for investment in hip fracture services. In exceptional circumstances, when demand exceeds capacity, risk-stratification tools such as the NHFS^23^ and CFS^24^ may guide prioritization. Higher-risk patients (NHFS ≥ 6, CFS ≥ 5) appear to be disproportionately affected by capacity delay.

This study had limitations. The robust methodological approach sought to minimize potential confounders, but its observational nature limits the ability to separate correlation from causation. Residual confounding cannot be excluded despite extensive adjustment and propensity-score matching. Furthermore, this was a single-center study, and organizational factors affect patient mortality after a hip fracture^30^. The definition of a delay in surgery as >36 hours was consistent with U.K. guidelines but varies internationally. Defining the time to surgery as starting at the time of presentation is consistent with most studies in the literature, but it is a proxy for the time from injury and thus introduces confounding when presentation following injury is delayed. The findings of this study must be interpreted with that limitation in mind. The actual time of injury is a critical variable but is rarely accurately recorded^13^. Costs in this study pertain only to the acute inpatient admission, and the greater costs and length of stay in the delayed group are likely due merely to their longer preoperative stay, rather than a difference between the groups. Finally, there was limited ethnic diversity, which reflects the local population but may limit generalizability.

In summary, this study highlights the importance of prioritizing timely surgery for patients with a hip fracture who are medically optimized for operative management. It further suggests that higher-risk and frailer patients may be priorities. Future studies should be multicentric to increase confidence in generalizability.

## Appendix

Supporting material provided by the authors is posted with the online version of this article as a data supplement at jbjs.org (https://links.lww.com/JBJS/J451).
